# Genetic architecture of Parkinson’s disease subtypes – Review of the literature

**DOI:** 10.3389/fnagi.2022.1023574

**Published:** 2022-10-20

**Authors:** Jarosław Dulski, Ryan J. Uitti, Owen A. Ross, Zbigniew K. Wszolek

**Affiliations:** ^1^Department of Neurology, Mayo Clinic, Jacksonville, FL, United States; ^2^Division of Neurological and Psychiatric Nursing, Faculty of Health Sciences, Medical University of Gdańsk, Gdańsk, Poland; ^3^Department of Neurology, St. Adalbert Hospital, Copernicus PL Ltd., Gdańsk, Poland; ^4^Department of Neuroscience, Mayo Clinic, Jacksonville, FL, United States

**Keywords:** genotype, phenotype, monogenic, oligogenic and polygenic inheritance, heterogeneity, genome-wide association study (GWAS), PD

## Abstract

The heterogeneity of Parkinson’s disease (PD) has been recognized since its description by James Parkinson over 200 years ago. The complexity of motor and non-motor PD manifestations has led to many attempts of PD subtyping with different prognostic outcomes; however, the pathophysiological foundations of PD heterogeneity remain elusive. Genetic contributions to PD may be informative in understanding the underpinnings of PD subtypes. As such, recognizing genotype-phenotype associations may be crucial for successful gene therapy. We review the state of knowledge on the genetic architecture underlying PD subtypes, discussing the monogenic forms, as well as oligo- and polygenic risk factors associated with various PD subtypes. Based on our review, we argue for the unification of PD subtyping classifications, the dichotomy of studies on genetic factors and genetic modifiers of PD, and replication of results from previous studies.

## Introduction

Parkinson’s disease (PD) is the second most prevalent neurodegenerative disorder after Alzheimer’s disease. The prevalence in the general population is low (0.3%); however, it increases rapidly with age and is estimated at 1 and 3% after 60 and 80 years, respectively (Balestrino and Schapira, 2020). Furthermore, among neurological disorders, PD displayed the most prominent growth in prevalence and disability in the last three decades (Ou et al., 2021). Most of the growing trend in PD incidence has been attributed to the aging of the population; however, other factors, including lifestyle choices, environmental pollution, occupational exposure, and genetic composition, and evolving treatments, are considered to play a role (Ou et al., 2021).

Traditionally, PD is characterized by four cardinal motor symptoms: bradykinesia, tremor, rigidity, and postural instability. However, patients differ in the severity of these symptoms, and distinct combinations of symptoms may predominate in the clinical picture. The heterogeneity of clinical presentations led to many attempts of PD subtyping. The research on PD subtypes evolved from studies on arbitrarily pre-defined groups into data-driven clustering of PD patients. The former (older) approach most often divides patients into tremor-dominant (TD)/indeterminate/postural instability and gait difficulty (PIGD) (Zetusky et al., 1985; Jankovic et al., 1990), TD/mixed/akinetic-rigid (AR) (Schiess et al., 2000; Korchounov et al., 2004; Kang et al., 2005) or TD/AR/gait difficulty (GD)/mixed (Konno et al., 2018) subtypes. In contrast, the latter (novel) approach is more advantageous in the research setting, as it allows hypothesis-free analysis of multiple domains; but it is rarely translated into an algorithm applicable in clinical practice (Fereshtehnejad et al., 2017; Lawton et al., 2018; Zhang et al., 2019; Mestre et al., 2021). To further confound the picture, non-motor symptoms have been recognized, including behavioral and mood disturbances, cognitive impairment, sensory symptoms, autonomic dysfunction, and sleep and wakefulness disorders (Dulski et al., 2015, 2018, 2019, 2022, forthcoming; Balestrino and Schapira, 2020; Siuda, 2021; Tipton, 2021). Table 1 presents the various PD subtypes investigated in the previous studies.

**TABLE 1:** Parkinson’s disease subtypes.

References	Number of subtypes	Parkinson’s disease subtypes	Classification basis
Zetusky et al. (1985) and Jankovic et al. (1990)	3	Tremor-dominant/indeterminate/postural instability and gait difficulty subtypes	Predominant motor symptoms
Schiess et al. (2000), Korchounov et al. (2004), Kang et al. (2005)	3	Tremor-dominant/mixed/akinetic-rigid subtypes	Predominant motor symptoms
Fereshtehnejad et al. (2017)	3	Mild motor-predominant/diffuse malignant/intermediate	Motor symptoms and non-motor symptoms (cognitive decline, RBD, and dysautonomia)
Lawton et al. (2018)	4	Fast motor progression/mild motor and non-motor disease/severe motor disease, poor psychological well-being and poor sleep/slow motor progression	Cognitive impairment, motor and non-motor symptoms
Konno et al. (2018)	4	Tremor-dominant/akinetic-rigid/gait difficulty/mixed subtypes	Predominant motor symptoms
Zhang et al. (2019)	3	Mild Baseline, Moderate Motor Progression/Moderate Baseline, Mild Progression/Severe Baseline, Rapid Progression	Motor and non-motor symptoms, CSF examination, DaTScan

CSF, cerebrospinal fluid; DaTscan, imaging of dopamine transporter using 123^*I*^-iofupane single photon emission computed tomography (SPECT); RBD, rapid eye movement (REM) sleep behavior disorder.

Having a first-degree relative with PD increases the risk by two-three fold; however, only 15% of patients have a positive family history of PD, and less than 10% are diagnosed with monogenic forms (Balestrino and Schapira, 2020; Hall et al., 2020). Furthermore, as of June 2022, over 100 genetic loci were associated with PD and other forms of parkinsonism; genetic contributions have been legion in the last decade (Dulski et al., forthcoming). Genetic studies are increasingly sophisticated and less costly, leading to more widespread application in research and clinical settings. In particular, with genome-wide association studies (GWAS), new variants or combinations of them (oligo- and polygenic inheritance) are being discovered.

In this manuscript, we review the state of knowledge on the genetic architecture underlying PD heterogeneity and subtypes. We summarize the phenotypes associated with mutations according to classification from our study on PD (TD/AR/GD/mixed subtypes) that we find most helpful in clinical settings (Konno et al., 2018). Finally, we provide implications of various genetic profiles for clinical management and suggest future research directions.

## Monogenic forms of Parkinson’s disease

Monogenic forms of PD may be inherited in an autosomal dominant, autosomal recessive, or X-linked manner, and account for approximately 30% of familial PD forms (Milanowski et al., 2021). Of note, single-gene forms of PD are not always familial, as a patient may have a recessive variant, incomplete penetrance, or *de novo* mutation; they are likely responsible for 3–5% of sporadic PD forms (Milanowski et al., 2021). Monogenic PD may present at different ages as juvenile-onset PD (JOPD) (below 20 years), early-onset PD (EOPD) (between 20 and 50 years), and late-onset PD (LOPD) (after 50 years) (Dulski et al., forthcoming). Table 2 presents characteristics of the monogenic forms of PD and their practical implications.

**TABLE 2:** Characteristics of the monogenic forms of Parkinson’s disease (PD) and their practical implications.

Gene	Mode of inheritance	Variant	Age of onset	Clinical features	Management implications
*DJ-1*	Autosomal recessive	Nucleotide substitutions	EOPD	Blepharospasm, lower limb dystonia common	Based on clinical symptoms
		Exonic and multiexonic deletions			
*LRRK2*	Autosomal dominant	p.Gly2019Ser	LOPD	Slight predilection toward GD and tremor; slow progression	Good response to L-Dopa, DBS, and LCIG. Careful use of dopamine agonists CASI (increased risk of sleep attacks and daytime sleepiness)
		p.Arg1441Gly	LOPD	TD at the disease onset (majority), followed by AR	
		p.Tyr1699Cys	LOPD	Atypical features (amyotrophy, dementia) in some patients	
*PINK1*	Autosomal recessive	Nucleotide substitutions	EOPD	Benign course, good response to L-Dopa, dementia rare	Based on clinical symptoms; good candidates for advanced therapies
		Exonic and multiexonic rearrangements (deletions and duplications)			
*PRKN*	Autosomal recessive	Structural (exonic rearrangements)	EOPD	Benign course, higher frequency of tremor, low asymmetry, good and sustained response to L-Dopa, dementia uncommon	Cautious use of dopamine agonists and CASI (increased risk of ICD). Ideal candidates for DBS and LCIG
		Nucleotide substitutions, small insertions/deletions			
*SNCA*	Autosomal dominant	Duplications	LOPD/EOPD	Heterogenous phenotype (from asymptomatic to severe), mild phenotype most common	Cautious use of anticholinergics, amantadine, and dopamine agonists. DBS and CASI are discouraged. LCIG seems to be the safest option
		Ala53Thr	LOPD/EOPD	Fast progression, low incidence of tremor, early emergence of L-Dopa complications	
		p. His50Gln	LOPD	TD, high risk of cognitive impairment	
		Triplications	EOPD	Rapid progression and atypical features	
		Nucleotide substitutions			
*VPS35*	Autosomal dominant	Asp620Asn	LOPD/EOPD	Low risk of atypical features, neuropsychiatric and cognitive problems	Based on clinical symptoms

AR, akinetic-rigid subtype; CASI, continuous apomorphine subcutaneous infusion; DBS, deep brain stimulation; EOPD, early-onset PD (between 20 and 50 years); GD, gait difficulty subtype; ICD, impulse control disorder; JOPD, juvenile-onset PD (below 20 years); LCIG, levodopa-carbidopa intestinal gel infusion; LOPD, late-onset PD (after 50 years); PD, Parkinson’s disease; TD, tremor-dominant subtype.

### Monogenic forms of Parkinson’s disease predominantly presenting with tremor-dominant subtype

#### LRRK2

Mutations in the Leucine-Rich Repeat Kinase 2 (*LRRK2*) gene are the most common cause of genetic PD and are responsible for 1% of sporadic and 5% of familial cases worldwide (Guadagnolo et al., 2021; Dulski et al., forthcoming). They are inherited in an autosomal dominant fashion. Most cases are of Caucasian descent (63%), followed by Hispanics (8%), and Asians (11%) (Trinh et al., 2018). The *LRRK2* gene encodes a mainly cytoplasmic protein involved in diverse biological activities, including neuronal vesicular trafficking, mitochondrial functions, and autophagy (Madureira et al., 2020; Guadagnolo et al., 2021; Lee et al., 2022). The phenotype resembles a classic LOPD, and at the clinical and neuropathological level, it cannot be distinguished from idiopathic PD (Trinh et al., 2018; Guadagnolo et al., 2021; Dulski et al., forthcoming). Numerous variants in the *LRRK2* have been reported; however, only some were associated with PD. At least seven variants (p.Arg1441Gly/Cys/His, p.Asn1437His, p.Gly2019Ser, p.Ile2020Thr, and p.Tyr1699Cys) are considered pathogenic, and a few others (p.Arg1628Pro, p.Gly2385Arg, and p.Ser1761Arg) are reported risk factors for PD (Guadagnolo et al., 2021; Simpson et al., 2022; Dulski et al., forthcoming). The most prevalent variant is p.Gly2019Ser, which alone may account for up 10–40% of the familial PD cases in the North African, Ashkenazi Jewish, and Mediterranean populations (Mirelman et al., 2013; Marras et al., 2016; Trinh et al., 2018; Guadagnolo et al., 2021). The p.Gly2019Ser variant and other studied *LRRK2* mutations display incomplete penetrance, increasing with age and impacted by other genetic variants and ethnical ancestry, with Northern Europeans developing symptoms later than Northern Africans (Mirelman et al., 2013; Hentati et al., 2014; Marras et al., 2016; Trinh et al., 2018; Guadagnolo et al., 2021). The p.Gly2019Ser variant has a heterogeneous presentation, but there seems to be slight predilection toward the postural instability, tremor, and generally a milder progression of motor and non-motor manifestations (including cognitive deterioration) (Mirelman et al., 2013; Marras et al., 2016; Trinh et al., 2018; Guadagnolo et al., 2021). The p.Arg1441Gly variant is manifested at the disease onset as a TD PD subtype in most patients, followed by an AR subtype (a quarter of patients), progression is slow, and dementia risk is low (Vinagre-Aragón et al., 2021). The p.Gly2385Arg variant predisposes to the PIGD subtype in the first few years of PD, faster motor progression, and cognitive decline (Marras et al., 2016). The p.Tyr1699Cys variant has various presentations, with atypical features (amyotrophy, dementia) in some patients (Kim et al., 2012). The *LRRK2* mutation carriers tend to respond well to dopaminergic therapy. In the advanced stages of the disease, they are good candidates for deep brain stimulation (DBS), and levodopa-carbidopa intestinal gel infusion (LCIG) (Trinh et al., 2018; Guadagnolo et al., 2021; Salles et al., 2021; Lee et al., 2022). However, the dopamine agonists and continuous apomorphine subcutaneous infusion (CASI) should be considered with caution, as *LRRK2* mutation carriers are at higher risk of sleep attacks and daytime sleepiness (Salles et al., 2021). And while LRRK2 neuropathology typically reflects “usual” PD pathology, with prominent synuclein inclusions and nigral cell loss, there may be significant variability in neuropathological findings leading some patients to be reclassified atypical PD despite having usual clinical presentations (Uitti et al., 2004). In summary, the *LRRK2* mutations are most often associated with TD and GD subtypes (Kim et al., 2012; Mirelman et al., 2013; Marras et al., 2016; Trinh et al., 2018; Madureira et al., 2020; Guadagnolo et al., 2021; Vinagre-Aragón et al., 2021; Lee et al., 2022; Simpson et al., 2022; Dulski et al., forthcoming).

### Monogenic forms of Parkinson’s disease predominantly presenting with akinetic-rigid subtype

#### PRKN

Mutations in the *PRKN* (Parkin) are the most common cause of EOPD, responsible for up to 15% of all PD cases with onset below 50 years old (Guadagnolo et al., 2021; Jia et al., 2022; Dulski et al., forthcoming). They are inherited in an autosomal recessive manner. Most patients were Asians (39%) and Caucasians (31%), followed by Hispanics (10%) (Kasten et al., 2018). The *PRKN* gene encodes a protein essential for mitochondrial homeostasis and mitophagy. Numerous pathogenic variants were reported, the most common being structural variants (exonic rearrangements), followed by missense and frameshift mutations (Dulski et al., forthcoming). The median age of onset is 31 years of age (Kasten et al., 2018). The phenotype resembles classic PD; however, it tends to have a more benign disease course, better responsiveness to levodopa, high frequency of bradykinesia, rigidity, tremor, and less asymmetry (Kasten et al., 2018; Guadagnolo et al., 2021; Jia et al., 2022; Dulski et al., forthcoming). Given that dementia, dysautonomia, and atypical features are uncommon and patients frequently develop fluctuations and dyskinesia, *PRKN*-PD patients are ideal candidates for DBS and infusion therapies (Kasten et al., 2018; Guadagnolo et al., 2021; Salles et al., 2021; Jia et al., 2022; Dulski et al., forthcoming). Of note, at least one study demonstrated that *PRKN*-PD patients might be more prone to impulse control disorder (ICD); therefore, caution should be used when administering dopamine agonists and CASI (Morgante et al., 2016). Approximately 2% of the population may carry one pathogenic variant in *PRKN*, and they seem to be at the same risk for developing PD as the non-carriers (Zhu et al., 2022; Dulski et al., forthcoming). In summary, carriers of *PRKN* mutations tend to manifest with an AR subtype.

#### PINK1

Pathogenic variants in the *PINK1* account for up to 5% of EOPD worldwide (Guadagnolo et al., 2021; Jia et al., 2022; Dulski et al., forthcoming). They are inherited in an autosomal recessive fashion. Most patients were Caucasians (33%), followed by mixed ethnicity (31%), and Asians (18%) (Kasten et al., 2018). The gene encodes a protein involved in mitochondrial functions. Numerous variants have been reported, with missense mutation responsible for nearly half of the cases (Guadagnolo et al., 2021; Jia et al., 2022; Dulski et al., forthcoming). The median age of onset is 32 years (Kasten et al., 2018; Guadagnolo et al., 2021; Jia et al., 2022; Dulski et al., forthcoming). The phenotype is similar to that of *PRKN*, but with a higher frequency of dystonia, dysautonomia, and a lower frequency of tremor (Kasten et al., 2018; Guadagnolo et al., 2021; Jia et al., 2022; Dulski et al., forthcoming). Cognitive impairment may be present but generally is rare (Kasten et al., 2018; Guadagnolo et al., 2021; Jia et al., 2022). The patients respond well to dopaminergic therapy and are good candidates for advanced treatment options (Kasten et al., 2018; Guadagnolo et al., 2021; Salles et al., 2021; Jia et al., 2022; Dulski et al., forthcoming). The studies on the carriers of one pathogenic variant did not show increased susceptibility for PD (Krohn et al., 2020; Dulski et al., forthcoming). In summary, *PINK1* mutations are most often linked with an AR subtype.

#### DJ-1

The mutations in the *DJ1* gene are explain up to 1% of EOPD cases (Guadagnolo et al., 2021; Jia et al., 2022; Dulski et al., forthcoming). They are inherited in an autosomal recessive manner. Most patients were of mixed ethnicity (38%), followed by Asiana (25%), Hispanics (13%), Ashkenazi Jews (13%), and Caucasians (13%) (Kasten et al., 2018). Like *PRKN* and *PINK1*, *DJ-1* encodes a protein crucial for the proper functioning of the mitochondria (Guadagnolo et al., 2021; Jia et al., 2022; Dulski et al., forthcoming). At least 27 pathogenic variants were reported, with missense mutations being the most common (Guadagnolo et al., 2021; Jia et al., 2022; Dulski et al., forthcoming). The median age of onset is 27 years (Kasten et al., 2018; Guadagnolo et al., 2021; Jia et al., 2022; Dulski et al., forthcoming). The phenotype is similar to that of *PRKN* and *PINK1*, with more frequent psychiatric symptoms (Kasten et al., 2018; Guadagnolo et al., 2021; Jia et al., 2022; Dulski et al., forthcoming). Blepharospasm and lower limb dystonia are also common (Kasten et al., 2018; Guadagnolo et al., 2021; Jia et al., 2022; Dulski et al., forthcoming). Most patients respond well to dopaminergic therapy; however, the data on this subject are limited (Kasten et al., 2018; Guadagnolo et al., 2021; Jia et al., 2022; Dulski et al., forthcoming). Therefore, every patient should be considered individually, with clinical symptoms determining the choice of oral and advanced therapies (Kasten et al., 2018; Guadagnolo et al., 2021; Salles et al., 2021; Jia et al., 2022; Dulski et al., forthcoming). In summary, *DJ-1* mutations are most often linked with an AR subtype.

### Monogenic forms of Parkinson’s disease predominantly presenting with mixed subtype

#### SNCA

Mutations in the *SNCA* (alpha-synuclein) gene are the second most prevalent cause of autosomal dominant PD and are responsible for 1–2% of autosomal dominant familial PD cases worldwide (Lesage et al., 2020). Most patients were Caucasians (70%), followed by Asians (16%) and Hispanics (14%) (Trinh et al., 2018). Pathogenic variants disrupt the lysosomal degradation of alpha-synuclein and lead to its aggregation (Guadagnolo et al., 2021). The most common are whole gene duplications, followed by a few missense mutations (most often p.Ala53Thr, but also p.Ala30Pro, p.Glu46Lys, p.Glu51Asp, p. Ala53Glu, and p. His50Gln) and triplications (Trinh et al., 2018; Lesage et al., 2020; Guadagnolo et al., 2021; Dulski et al., forthcoming). The *SNCA* duplications have a heterogeneous phenotype, including asymptomatic status at the one end of the spectrum, benign phenotype with slow progression in the middle (most common), and rapidly progressive parkinsonism on the opposite end of the spectrum (rare) (Guadagnolo et al., 2021). The p.Ala53Thr variant manifests with classic PD, albeit the TD PD subtype is infrequent and is associated with faster disease progression and early emergence of levodopa complications (Guadagnolo et al., 2021). The p. His50Gln often manifests as a TD PD subtype, and cognitive impairment is common (Appel-Cresswell et al., 2013; Guadagnolo et al., 2021). The other mutations are very rare and there is insufficient data to draw genotype-phenotype conclusions. The *SNCA* triplications present with EOPD with a rapid progression and atypical features. Generally, *SNCA*-related PD is associated with a high rate of psychotic symptoms and depression, early onset of cognitive decline, and autonomic dysfunction (Guadagnolo et al., 2021). Therefore, anticholinergics, amantadine, and dopamine agonists should be limited in these patients. In addition, these patients are at high risk of adverse effects related to DBS and CASI, and LCIG seems to be the safest option (Salles et al., 2021). In summary, *SNCA* mutations are most often associated with a mixed subtype (Trinh et al., 2018; Lesage et al., 2020; Guadagnolo et al., 2021; Salles et al., 2021; Dulski et al., forthcoming).

### Monogenic forms of Parkinson’s disease without clear predilection toward a specific subtype

#### Autosomal dominant forms of monogenic Parkinson’s disease

Until now, mutations in several other genes have been in autosomal dominant familial PD. This gene list includes, but is not limited to, *VPS35, EIF4G1, TMEM230, CHCHD2, LRP10, RIC3, ARSA*, and *PSAP* (Guadagnolo et al., 2021; Dulski et al., forthcoming).

Pathogenic variants in the *VPS35* gene are responsible for less than 1% of familial PD cases worldwide (Trinh et al., 2018; Guadagnolo et al., 2021; Dulski et al., forthcoming). They are inherited in an autosomal dominant manner. Most patients were Caucasians (45%), followed by Asians (35%) and Ashkenazi Jews (20%) (Trinh et al., 2018). The *VPS35* gene encodes the protein involved in the endosome-trans-Golgi network trafficking (Trinh et al., 2018; Guadagnolo et al., 2021; Dulski et al., forthcoming). At least 11 variants were reported, but until now, pathogenicity was confirmed only for the p.Asp620Asn variant (Trinh et al., 2018; Guadagnolo et al., 2021; Dulski et al., forthcoming). The phenotype resembles classic PD with a mean age of onset at 50; however, a milder course, with a low risk of atypical features, neuropsychiatric and cognitive problems, was noted (Trinh et al., 2018; Guadagnolo et al., 2021; Dulski et al., forthcoming). The reports on the prevalence of tremor, postural instability, dysautonomia, and daytime sleepiness provided inconsistent results and seemed similar to sporadic PD (Trinh et al., 2018; Liu and Le, 2020; Guadagnolo et al., 2021; Salles et al., 2021; Dulski et al., forthcoming). Most studies reported a good response to levodopa. Therefore, the patient’s management should be tailored to clinical findings. Based on previous studies, no conclusion can be drawn regarding the most often associated phenotype (Trinh et al., 2018; Liu and Le, 2020; Guadagnolo et al., 2021; Salles et al., 2021; Dulski et al., forthcoming).

Mutations in other genes were reported only in a few families with PD, and the causative role of some of them in PD is debated. In particular, recent studies suggest that *EIF4G1* may not play a role in PD (Saini et al., 2021). Pathogenic variants in these genes may present as LOPD (*EIF4G1, TMEM230, CHCHD2, LRP10, RIC3, ARSA, PSAP)*, EOPD *(CHCHD2, RIC3, ARSA*, and *PSAP)*, and JOPD *(ARSA). LRP-10*-associated phenotype is often accompanied by dementia, which may be the first presentation (Guadagnolo et al., 2021; Dulski et al., forthcoming). Patients with PD due to the *ARSA* mutations often present with a tremor in their youth and develop TD PD in their 5th–6th decade (Dulski et al., forthcoming). Mutations in these genes may manifest as classic PD with a good and long-lasting response to dopaminergic therapy. Due to the scarcity of information on the genotype-phenotype associations, the therapy should be tailored based on clinical symptomatology. The advanced treatment options should also be considered (Guadagnolo et al., 2021; Saini et al., 2021; Dulski et al., forthcoming).

#### Autosomal recessive forms of monogenic Parkinson’s disease

At least seven other known genetic loci are associated with autosomal recessive parkinsonism, including *ATP13A2* (Kufor-Rakeb syndrome), *PLA2G6* (neurodegeneration with brain iron accumulation 2A), *FBXO7*, *DNAJC6*, *SYNJ1*, *VPS13C*, and *WASL* (Kumar et al., 2021; Dulski et al., forthcoming). Mutations in these genes present as JOPD (*ATP13A2*, *PLA2G6*, *FBXO7*, *DNAJC6*, and *SYNJ1*) or EOPD (*VPS13C* and *WASL*) (Kumar et al., 2021; Dulski et al., forthcoming). The *WASL*-associated phenotype is that of slowly progressive PD with a good response to levodopa (Kumar et al., 2021; Dulski et al., forthcoming). However, the pathogenic variants in the other genes usually lead to PD accompanied by atypical features, the onset of dementia early in the disease course, fast progression, and variable response to levodopa (Kumar et al., 2021; Wittke et al., 2021; Dulski et al., forthcoming). Hence, except for *WASL*-related PD, the treatment should mainly consist of oral pharmacological therapy, preferentially with levodopa. In *WASL*-associated PD, DBS and other advanced therapies may be considered.

#### X-linked forms of Parkinson’s disease

Mutations in at least eight genetic loci (*TAF1, FMR1, RAB39B, WDR45*, *GLA*, *MeCP2*, *PGK1*, and *ATP6AP)* on the X chromosome were associated with the development of PD (Di Lazzaro et al., 2021; Dulski et al., forthcoming). Contrary to the autosomal monogenic forms, pathogenic variants on the X chromosome usually manifest at a young age (JOPD or EOPD), with parkinsonism accompanied by many (atypical) features including intellectual disability or early cognitive decline, psychiatric features, spasticity, seizures (Di Lazzaro et al., 2021; Dulski et al., forthcoming), myoclonus, dystonia, and usually display a poor response to dopaminergic treatment. Most of the affected individuals are males; however, rarely, female carriers may present with a mild phenotype of pure PD with a good response to levodopa and no atypical features (Di Lazzaro et al., 2021; Dulski et al., forthcoming). Based on previous studies, no conclusion regarding the most often associated PD subtypes can be drawn.

## Genetic risk factors associated with Parkinson’s disease symptomatology

Table 3 summarizes the genetic factors *associated* with PD symptomatology.

**TABLE 3:** The genetic risk factors associated with Parkinson’s disease (PD) symptomatology.

Gene	Variant/polymorphism/haplotype	Prevalence	Clinical features	Management implications
*GBA*	More than 130 variants linked with PD. Most common: N370S p.E326K p.T369M p. L444P	2–30%	AR or GD subtype; faster progression, high burned of non-motor symptoms and non-dopaminergic symptoms, more prone to cognitive decline and other neuropsychiatric symptoms, worse prognosis	DBS discouraged; cautious use of anticholinergics and dopamine agonists; therapy with LCIG in advanced cases seem to be the safest option
*MAPT*	H1 haplotype and its subtypes	80%	Increased risk of PD and other neurodegenerative disorders	Not yet established
*APOE*	E4	Up to 20%	Increased risk of cognitive decline	Lifestyle changes, managing metabolic and vascular risks
*COMT*	rs4680 (A;A)	Common	Low enzyme activity (presumably higher dopamine levels)	Higher risk of polyneuropathy and L-Dopa induced dyskinesia,
	rs4680 (G;G)		High enzyme activity (presumably lower dopamine levels)	May require higher L-Dopa doses
	rs4646318	Common	Polymorphism linked with ICD	Not yet established
*MAOB*	Many polymorphisms impacting the *MAOB* activity	Common	Low enzyme activity	Higher risk of L-Dopa-induced dyskinesia
			High enzyme activity	Male patients may require higher L-Dopa doses
*DRD1*	rs4532	Common	Linked with ICD	Not yet established
	rs4867798	Common		
*DRD 2*	rs1800497	Common		
*DRD3*	rs6280	Common		
*DRD3*	rs6280	Common	Linked with susceptibility to diphasic dyskinesia	Not yet established
*SLC6A3*	rs3836790	Common	Associated with response to L-Dopa	Not yet established
	rs28363170			
*DDC*	rs921451	Common	Linked with response to L-Dopa	Not yet established
	rs4490786	Common	Linked with ICD	Not yet established
*MTHFR*	rs1801133 (C;T) (T;T)	Common	Hyperhomocysteinemia	Increased monitoring for hyperhomocysteinemia and related complications
	rs1801131 (A;C) (C;C)			
*GRIN2B*	rs7301328	Common	Linked with ICD	Not yet established
*HTR2A*	rs6313			
*OPRK1*	rs702764			
*OPRM1*	rs677830			
*TPH2*	rs4290270			

AR, akinetic-rigid subtype; CASI, continuous apomorphine subcutaneous infusion; DBS, deep brain stimulation; ICD, impulse control disorder; LCIG, levodopa-carbidopa intestinal gel infusion; PIGD, postural instability and gait difficulty subtype; PD, Parkinson’s disease; TD, tremor-dominant subtype.

### Genetic risk factors for akinetic-rigid/gait difficulty subtypes

#### GBA

Biallelic pathogenic variants in the *GBA* gene were classically linked with Gaucher’s disease, an autosomal recessive multisystem disorder with varied neurological manifestations. In recent years, heterozygous variants in *GBA* were found to be the most common genetic risk factors for PD, with an estimated prevalence of 2–30% worldwide (Jesús et al., 2016; Iwaki et al., 2019b; Petrucci et al., 2020; Straniero et al., 2020; Menozzi and Schapira, 2021; Dulski et al., forthcoming). The penetrance is low, and over the lifespan is estimated at 9.1%; however, it increases with age up to 30% at 80 years (Jesús et al., 2016; Iwaki et al., 2019b; Petrucci et al., 2020; Straniero et al., 2020; Menozzi and Schapira, 2021; Dulski et al., forthcoming). As penetrance is higher in carriers with a positive family history of PD than without such history, other genetic factors may be at play (Jesús et al., 2016; Iwaki et al., 2019b; Petrucci et al., 2020; Straniero et al., 2020; Menozzi and Schapira, 2021; Dulski et al., forthcoming).

More than 130 genetic variants associated with PD have been described (Jesús et al., 2016; Iwaki et al., 2019b; Petrucci et al., 2020; Straniero et al., 2020; Menozzi and Schapira, 2021; Dulski et al., forthcoming). Generally, heterozygote status increases the risk for PD by five times (Jesús et al., 2016; Iwaki et al., 2019b; Petrucci et al., 2020; Straniero et al., 2020; Menozzi and Schapira, 2021; Dulski et al., forthcoming). In addition, the *GBA* mutations worsen the prognosis of PD (Jesús et al., 2016; Lythe et al., 2017; Iwaki et al., 2019b; Mangone et al., 2020; Petrucci et al., 2020; Straniero et al., 2020; Menozzi and Schapira, 2021; Pal et al., 2022; Dulski et al., forthcoming). Carriers of *GBA* mutations develop PD a few years earlier, more often they present with the AR or PIGD subtype, tend to progress faster, and have a higher burden of non-non-motor symptoms (Jesús et al., 2016; Lythe et al., 2017; Iwaki et al., 2019b; Mangone et al., 2020; Petrucci et al., 2020; Straniero et al., 2020; Menozzi and Schapira, 2021; Pal et al., 2022; Dulski et al., forthcoming). In particular, cognitive decline and other neuropsychiatric symptoms are common and occur earlier than in non-carriers (Jesús et al., 2016; Lythe et al., 2017; Iwaki et al., 2019b; Mangone et al., 2020; Petrucci et al., 2020; Straniero et al., 2020; Menozzi and Schapira, 2021; Pal et al., 2022; Dulski et al., forthcoming). Motor non-dopaminergic symptoms, like dysphagia, dysarthria and freezing of gait were also reported to be more common (Jesús et al., 2016; Lythe et al., 2017; Iwaki et al., 2019b; Mangone et al., 2020; Petrucci et al., 2020; Straniero et al., 2020; Menozzi and Schapira, 2021; Pal et al., 2022; Dulski et al., forthcoming). However, individual variants harbor the different risks of developing PD and have a diverse impact on the disease course (Jesús et al., 2016; Lythe et al., 2017; Iwaki et al., 2019b; Mangone et al., 2020; Petrucci et al., 2020; Straniero et al., 2020; Menozzi and Schapira, 2021; Pal et al., 2022; Dulski et al., forthcoming). The four missense variants (p.N370S, p.E326K, p.T369M, and p. L444P) account for more than 80% of *GBA* mutations associated with PD (Jesús et al., 2016; Lythe et al., 2017; Iwaki et al., 2019b; Mangone et al., 2020; Petrucci et al., 2020; Straniero et al., 2020; Menozzi and Schapira, 2021; Pal et al., 2022; Dulski et al., forthcoming). The p.L444P is considered the most deleterious, showing the strongest link with PD and dementia, the highest penetrance, and the mean age of disease onset of 51 years (Jesús et al., 2016; Lythe et al., 2017; Iwaki et al., 2019b; Mangone et al., 2020; Petrucci et al., 2020; Straniero et al., 2020; Menozzi and Schapira, 2021; Pal et al., 2022; Dulski et al., forthcoming). The p.N370S is the most common variant representing more than 70% of *GBA* mutations in certain populations (i.e., Ashkenazi Jewish) (Jesús et al., 2016; Lythe et al., 2017; Iwaki et al., 2019b; Mangone et al., 2020; Petrucci et al., 2020; Straniero et al., 2020; Menozzi and Schapira, 2021; Pal et al., 2022; Dulski et al., forthcoming). The p.N370S is also linked with earlier age of onset (mean of 54 years), higher rate of dementia, and intermediate penetrance (Jesús et al., 2016; Lythe et al., 2017; Iwaki et al., 2019b; Mangone et al., 2020; Petrucci et al., 2020; Straniero et al., 2020; Menozzi and Schapira, 2021; Pal et al., 2022; Dulski et al., forthcoming). The p.T369M is the most benign, with the lowest penetrance (2.15%), low risk of dementia, and the normal age of PD onset (Jesús et al., 2016; Lythe et al., 2017; Iwaki et al., 2019b; Mangone et al., 2020; Petrucci et al., 2020; Straniero et al., 2020; Menozzi and Schapira, 2021; Pal et al., 2022; Dulski et al., forthcoming). The previous studies provided inconsistent data on the effects of p.E326K; however, recently, it was shown to confer a higher risk of PIGD subtype, faster motor progression, and a higher rate of dementia than expected (Jesús et al., 2016; Lythe et al., 2017; Iwaki et al., 2019b; Mangone et al., 2020; Petrucci et al., 2020; Straniero et al., 2020; Menozzi and Schapira, 2021; Pal et al., 2022; Dulski et al., forthcoming). Other *GBA* variants (including p.E365K, p.T408M) were also linked with faster motor and cognitive progression (Jesús et al., 2016; Lythe et al., 2017; Iwaki et al., 2019b; Mangone et al., 2020; Petrucci et al., 2020; Straniero et al., 2020; Menozzi and Schapira, 2021; Pal et al., 2022; Dulski et al., forthcoming).

Given the preponderance of neuropsychiatric features, anticholinergics and dopamine agonists should be used with caution in the GBA carriers. Even more, caution should be exercised when considering therapy with DBS. A few studies have demonstrated that *GBA* mutation carriers are at higher risk of cognitive decline following DBS and thus a worse outcome (Lythe et al., 2017; Mangone et al., 2020; Pal et al., 2022). In light of these findings, therapy with LCIG in advanced PD with levodopa complications seems to be the safest option.

In summary, *GBA* mutations are most often associated with AR and GD subtypes.

#### MAPT

Pathogenic variants in *MAPT*, encoding microtube-associated protein tau, were described in various forms of inherited neurodegenerative disorders (Paul et al., 2016; Deutschlander et al., 2020; Dulski et al., forthcoming). Most of them manifested with autosomal dominant atypical parkinsonisms and dementia; however, in rare cases, they presented as classic PD (Paul et al., 2016; Deutschlander et al., 2020; Dulski et al., forthcoming). Recently, the *MAPT* locus was divided into two main haplotypes, H1 and H2 (Paul et al., 2016; Deutschlander et al., 2020; Dulski et al., forthcoming). The H1 haplotype is the most common, occurring in more than 80% of the general population, and associated with an increased risk of PD, atypical parkinsonisms, Alzheimer’s disease, and multiple other neurodegenerative diseases (Paul et al., 2016; Deutschlander et al., 2020; Dulski et al., forthcoming). On the contrary, the rare haplotype H2 seems to have a protective effect over neurodegeneration (Paul et al., 2016; Deutschlander et al., 2020; Dulski et al., forthcoming). In line with this, patients with TD-PD tend to have a higher prevalence of H2 haplotype than other PD subtypes (non-tremor dominant), which, by contrast, are more likely to carry H1 haplotype (Pascale et al., 2016). The haplotype H1 was further divided into 16 subtypes. Patients with PD and H1j subhaplotype had a lower risk of tremor and were more prone to rapid eye movement (REM) sleep behavior disorder (RBD), whereas H1r conferred a lower risk of bradykinesia, and H1b increased the risk for orthostatic hypotension (Deutschlander et al., 2020).

### Genetic risk factors for complications of levodopa therapy

The *COMT* and *MAOB* genes encode two major enzymes involved in catecholamine metabolism. Most studies focused on the impact of *COMT* polymorphisms resulting in low, medium, and high COMT activity (Bialecka et al., 2008; Andréasson et al., 2017; Erga et al., 2018; Sampaio et al., 2018; Falla et al., 2021; Soraya et al., 2022). Some studies showed that PD patients with high COMT activity require higher levodopa doses and are at and lower risk of levodopa-induced dyskinesia, compared to the PD patients with low COMT activity. However, other studies provided contradictory results concerning the dyskinesia risk and COMT activity (Bialecka et al., 2008; Andréasson et al., 2017; Erga et al., 2018; Sampaio et al., 2018; Falla et al., 2021; Soraya et al., 2022). One *COMT* polymorphism (rs4646318) was found linked with impulse control disorder in PD (Erga et al., 2018). Of note, PD patients with low COMT activity may be at increased risk of developing polyneuropathy compared to those with high COMT activity (Andréasson et al., 2017). Only a few studies investigated the impact of MAO-B polymorphisms and PD symptomatology. Patients with MAOB polymorphism associated with a lower enzyme activity are more susceptible to levodopa-induced dyskinesia (Sampaio et al., 2018). In addition, male patients with polymorphisms resulting in high MAO-B activity tend to be treated with higher levodopa doses (Sampaio et al., 2018).

Dopamine D receptors 1–5 are encoded by the *DRD* 1–5 genes. Most of the previous studies focused on the *DRD* 1–3 genes (Lee et al., 2011; Zainal Abidin et al., 2015; Miller et al., 2018; Li et al., 2022). The *DRD3* rs6280 (T > C) polymorphism was linked with higher susceptibility for diphasic dyskinesia (Lee et al., 2011). Another polymorphism within the *DRD2*, rs1076560 (G > T) was associated with gait dysfunction and better gait improvement in dopaminergic treatment compared to homozygous G allele carriers (Miller et al., 2018).

The *SLC6A3* gene encodes for the dopamine transporter (DAT). The *SLC6A3* polymorphisms (rs3836790 and rs28363170) were associated with motor response to levodopa treatment. However, some of the other studies did not confirm this observation (Moreau et al., 2015; Li et al., 2020, 2022; Soraya et al., 2022).

The polymorphisms in the *DDC* gene, coding for L-amino acid decarboxylase (AAAD), were investigated only in a few studies concerning PD (Moreau et al., 2015; Erga et al., 2018; Li et al., 2020; Soraya et al., 2022). In one recent study, the *DDC* polymorphism (rs921451) was found to modulate the motor response to levodopa; however, it was not confirmed in other studies (Moreau et al., 2015; Li et al., 2020).

The mutations in the *MTHFR* gene (rs1801133, C > T; rs1801131, A > C) are a known cause of hyperhomocysteinemia in the general population (Szadejko et al., 2016; Liu et al., 2018). Furthermore, hyperhomocysteinemia was associated with an increased risk of polyneuropathy and cardiovascular disease (Białecka et al., 2012; Szadejko et al., 2016; Liu et al., 2018). Since levodopa may increase homocysteine levels, PD patients have a particularly high risk of hyperhomocysteinemia. In addition, rs1801131 and rs1801133 *MTHFR* polymorphisms were linked with an increased risk of developing PD (Szadejko et al., 2016; Liu et al., 2018). However, recent metanalysis could not replicate the proposed association between rs1801131 and rs1801133 *MTHFR* polymorphisms and higher risk of developing PD (Diao et al., 2019). Screening PD patients treated with LCIG or high doses of levodopa for *MTHFR* mutations may select a subpopulation of patients with the highest risk of hyperhomocysteinemia-related complications, enabling monitoring this subpopulation and choosing optimal pharmacological therapy.

### Genetic risk factors for cognitive decline

*GBA* variants seem to be the most prevalent genetic risk factors modifying the susceptibility to developing cognitive decline in PD patients. However, variants in *APOE* and *MTHFR* were also linked with a higher propensity for cognitive decline. The *APOE* gene codes for Apolipoprotein E and occurs in three alleles, E2, E3, and E4. The E3 is the most prevalent and has a frequency of approximately 80%. The E4 may be found in up to 20% of the population and was associated with a higher risk of Alzheimer’s dementia (AD), whereas the rarest E2 was linked with a lower risk of cognitive decline (Paul et al., 2016; Iwaki et al., 2019b; Dilliott et al., 2021; Tipton et al., 2021; Counsell et al., 2022; Li et al., 2022). Previous studies have demonstrated that patients with PD and at least one E4 allele are at increased risk of cognitive deterioration (Paul et al., 2016; Iwaki et al., 2019b; Dilliott et al., 2021; Tipton et al., 2021; Counsell et al., 2022; Li et al., 2022). However, contrary to AD, the protective effect of E2 on cognition was not confirmed in PD, and some showed that it might even increase the risk of PD and atypical parkinsonisms (Dilliott et al., 2021; Li et al., 2022). The *MTHFR* gene and its implications and in PD were discussed in-depth above. Nonetheless, as hyperhomocysteinemia was linked with an increased risk of cognitive decline, PD patients carrying an *MTHFR* mutation (rs1801133, C > T; rs1801131, A > C) are more prone to developing dementia (Białecka et al., 2012; Liu et al., 2018).

### Genetic risk factors for impulse control disorder

Several polymorphisms of the *DRD* 1 (rs4532, C > T; rs4867798, T > C), *DRD* 2 (rs1800497, C > T), *DRD* 3 rs6280 (T > C) were associated with an increased risk of impulsive behaviors in PD patients (Zainal Abidin et al., 2015).

Other studies demonstrated that *DDC* (rs4490786), *GRIN2B* (rs7301328), *HTR2A* (rs6313), *OPRK1* (rs702764), *OPRM1* (rs677830), and *TPH2* (rs4290270) polymorphisms were also associated with ICD in PD patients (Lee et al., 2011, 2012; Zainal Abidin et al., 2015; Erga et al., 2018; Miller et al., 2018; Li et al., 2022).

## Oligo- and polygenic genetic factors behind Parkinson’s disease and its heterogeneity

Table 4 summarizes the previous genetic studies on PD heterogeneity.

**TABLE 4:** Previous genetic studies on Parkinson’s disease (PD) heterogeneity.

Study	Population	Design	Gene	Variant/polymorphism	Clinical features
Cooper et al. (2017)	1,040 PD patients	Evaluation of 10 SNPs associated with PD risk	*SNCA*	rs356182 (G;G)	Linked with TD phenotype, a slower rate of motor progression, and lower SNCA expression in the cerebellum
Blauwendraat et al. (2019)	28,568 PD patients	GWAS	*SNCA*	rs356203	Association with age of onset of PD
			*TMEM175*	rs34311866	
Iwaki et al. (2019b)	4,307 PD patients	GWAS	*GBA*	p.E365K	Faster cognitive decline and motor progression; higher susceptibility to RBD
				p.N370S	Faster motor progression; higher susceptibility to dyskinesia and motor fluctuations; higher risk of daytime sleepiness
				p.T408M	Faster motor progression; higher risk of RBD
			*LRRK2*	rs76904798	Linked with faster motor progression
			*PMVK*	rs114138760	Higher susceptibility to developing wearing off
Iwaki et al. (2019a)	4,093 PD patients	GWAS	*SLC44A1*	rs382940	Linked with a risk of motor progression.
			*intergenic variant close to LRRK2*	rs76904798	
			*intergenic variant*	rs61863020	Associated with risk of insomnia
			*CTSB*	rs1293298	
			*NOD2*	rs6500328	Associated with incidence of daytime slepiness
			*intergenic variant close to KCNS3*	rs76116224	
			*intergenic variant close to KRTCAP2*	rs35749011	Associated with the risk of cognitive impairment
			*GBA*	p.T408M	Linked with the higher risk of faster motor progression
				p.E365K	Linked with the higher risk of cognitive impairment
Szwedo et al. (2020)	433 PD patients and 417 controls	Evaluation of 5 variants within *SNCA*	*SNCA*	rs356219 (G;G)	Slightly faster cognitive decline on long-term follow-up (9 years)
				rs2870004, rs5019538, rs763443, and rs356182	No impact
Alfradique-Dunham et al. (2021)	3,212 PD patients	GWAS	No significant association between common variants and PD motor subtypes (TD/PIGD)		
			*GPNMB*	rs199351	Suggestive associations with the motor phenotype (TD vs. PIGD)
			*SH3GL2*	rs10756907	
			*HIP1R*	rs10847864	
			*RIT2*	rs12456492	
			*FBRSL1*	rs11610045	
			*STK32B*	rs2301857	
König et al. (2021)	231 PD patients	WES association analysis	*OPRM1*	rs1799971	Linked with time to L-Dopa-induced dyskinesia onset
			*MAD2L2*	rs2233019	
			*MAP7*	rs35350783	
Tan et al. (2021)	3,364 PD patients	GWAS	*APOE*	ε4 allele	Associated with faster cognitive decline
			*ATP8B2*	–	Faster motor progression
Liu et al. (2021)	3,821 PD patients	GWAS	*RIMS2*	rs182987047 (T)	Linked with risk of dementia in PD
			*TMEM108*	rs138073281	
			*WWOX*	rs8050111	
			*GBA*	L444P and other pathogenic mutations	
			*APOE*	ε4 allele	
Weintraub et al. (2022)	5,770 PD patients	GWAS	*DAB1*	rs148267997	Linked with susceptibility to ICD
			*PRKAG2*	rs2302532)	
			*MEFV*	s11466021	
			*PRKCE*	rs78448334	
Visanji et al. (2022)	150 PD patients	GWAS	*CRHR1*	rs75638861	Increased risk of axial symptoms aggravation following DBS
			*IP6K2*	–	
			*PRSS3*	rs142022985	
Grover et al. (2022)	8,535 PD patients	GWAS	*TMEM175*	rs34311866	Associated with the age of PD onset
			*SNCA*	rs983361	
			*BST1*	rs4698412	

GWAS, genome-wide association study; ICD, impulse control disorder; PD, Parkinson’s disease; PIGD, postural instability and gait difficulty subtype; RBD, rapid eye movement (REM) sleep behavior disorder; SNP, single nucleotide polymorphism; TD, tremor-dominant subtype; WES, whole exome sequencing.

In 2019, the largest GWAS in PD, involving almost 38 thousand patients, found 90 common independent variants, which together accounted for 16–36% of the observed PD heritability (Nalls et al., 2019). Recently, a large meta-analysis on PD GWAS on 8,535 patients revealed that polymorphisms of the *TMEM175* (rs34311866), *SNCA* (rs983361), and *BST1* (rs4698412) correlated with the age of the disease onset (Grover et al., 2022).

The rs356182 polymorphism near the *SNCA* gene was linked with the TD/PIGD phenotype. The GG genotype correlated with TD subtype, showed a slower rate of motor progression, and had lower *SNCA* expression in the cerebellum (Cooper et al., 2017). However, in another study, *SNCA* polymorphisms (rs356219, rs2870004, rs5019538, rs763443, and rs356182) that were previously linked with PD risk, were found not to contribute significantly to PD heterogeneity (Szwedo et al., 2020). A large GWAS study on 28,568 PD patients found that two genetic loci (*SNCA, TMEM175*) were modulating the age of the disease onset (Blauwendraat et al., 2019). In 2019, a GWAS study on 4,307 PD patients found that *GBA* variants (p.E365K, p.N370S, and p.T408M) correlated with rate of cognitive and motor progression, risk of developing dyskinesia, motor fluctuations, RBD, and daytime sleepiness (Iwaki et al., 2019b).

Another GWAS study in 2019, on 4,039 PD patients confirmed the association of GBA variants with higher risk of cognitive impairment (p.E365K) and faster motor progression (p.T408M), as well as, found a few genetic loci linked with the risk of sleep disturbances (rs61863020, rs1293298, rs6500328, and rs76116224), worsening of motor symptoms (rs382940, rs382940) and cognitive decline (rs35749011) (Iwaki et al., 2019a). However, only two polymorphisms (rs382940 in *SLC44A1*, and rs61863020 – intergenic variant) reached the genomewide significance (Iwaki et al., 2019a). The same study found that the T allele of rs76904798 polymorphism within *LRRK2* increased susceptibility to motor progression, and rs114138760 within *PMVK* was linked with the risk of developing wearing off (Iwaki et al., 2019b). Two large studies from 2021, confirmed that PD patients with *APOE* E4 allele or *GBA* variants are at increased risk of faster cognitive deterioration and identified novel polymorphism linked with faster cognitive (*RIMS2*, *TMEM108*, and *WWOX*) and motor (*ATP8B2*) progression (Liu et al., 2021; Tan et al., 2021). Another GWAS study in 2021, on 3,212 PD cases, did not find any significant association between common variants and PD motor subtypes (TD/PIGD) (Alfradique-Dunham et al., 2021). However, the authors reported multiple suggestive associations, including the polymorphisms of *GPNMB* (rs199351), *SH3GL2* (rs10756907), *HIP1R* (rs10847864), *RIT2* (rs12456492), *FBRSL1* (rs11610045), and *STK32B* (rs2301857) genes (Alfradique-Dunham et al., 2021). Interestingly, a variant in the *STK32B* was previously reported in other GWAS as a risk factor for essential tremor (Müller et al., 2016). Whole-exome sequencing (WES) association analysis of 231 PD patients found the polymorphisms in the *OPRM1* (rs1799971), *MAD2L2* (rs2233019), and *MAP7* (rs35350783) genes to be linked with time to levodopa-induced dyskinesia onset (König et al., 2021). In 2022, GWAS on 5,770 patients with PD detected polymorphisms of *DAB1* (rs148267997), *PRKAG2* (rs2302532), *MEFV* (rs11466021), and *PRKCE* (rs78448334) genes associated with impulse control disorders (Weintraub et al., 2022). In 2022, a study on 150 PD patients treated with DBS found associations between the deterioration of axial symptoms following surgery and polymorphisms of *CRHR1 (rs75638861)*, *IP6K2* (SNP not listed), and *PRSS3 (rs142022985)* genes (Visanji et al., 2022).

## Discussion

The majority of studies on PD subtypes used the data-driven (hypothesis-free) approach to uncover phenotypic clusters. The most frequently assessed phenotypic domains were the motor features, cognitive functions, and other neuropsychiatric symptoms. Nevertheless, despite being superseded by the data-driven clustering in the research, the PD subtyping, based mainly on studies on pre-defined (hypothesis-driven) motor phenotypes, is still the one most commonly used in everyday clinical practice. Neurologists thoroughly consider the patient’s motor predominant symptomatology, which significantly influences management strategy. Levodopa is the most effective medication for PD and is considered the gold standard against which other drugs and therapies are compared. Levodopa is the mainstay of PD therapy in most patients; however, in patients with certain phenotypes, other treatments should also be considered.

In patients with the GD subtype, an improvement on levodopa alone is often not satisfactory, and additional medications are given to improve gait and balance. In clinical settings, amantadine and rivastigmine are most often used (Cui and Lewis, 2021). Patients with advanced PD, motor fluctuations, and levodopa-induced dyskinesia are good candidates for DBS and infusional therapies. In such cases, the phenotype (mainly motor symptoms, cognitive status, and autonomic functions) guides the choice of therapies. Additionally, the predominant symptomatology determines the type of physical therapy, selection of assistive devices, and others (Dijk et al., 2020). Nevertheless, the clinical implications of PD subtypes go beyond the choice of therapy. Previous studies consistently demonstrated that different motor subtypes vary in disease progression and the burden of non-dopaminergic (e.g., cognitive decline, dysautonomia, RBD) symptoms. The TD subtype is considered the phenotype with a lower risk of cognitive decline, slower progression, and lesser impact of non-dopaminergic symptoms compared to GD and AR subtypes (Marras et al., 2020; Ren et al., 2020, 2021; Kohat et al., 2021; Myers et al., 2021; Lee et al., 2022). Lastly, the PD subtyping is relevant for clinical trials, as some protocols include the presence of specific phenotypes in the inclusion or exclusion criteria. Notably, that may be relevant to gene therapy, which raised high hopes among PD patients and physicians (Fiandaca et al., 2020). Taken together, determining the subtype of individual PD patients is of great importance for the time being and long-term prognosis.

Nonetheless, some studies demonstrated that patients might change their phenotypes over time, whereas most did not provide longitudinal data (Kohat et al., 2021; Mestre et al., 2021). Thus, the stability of PD subtypes over time is largely unknown, and there is a need to find markers that predict the susceptibility of a patient to develop certain symptoms. Therefore, in the PD subtyping research, high expectations were placed on studies investigating the biomarkers of the disease progression. However, the biological underpinnings of PD are complex and not completely understood. The neurodegenerative process is characterized by progressive loss of dopaminergic neurons in the pars compacta of substantia nigra, and misfolding and aggregation of α-synuclein in the cytoplasm of the neurons (Lewy bodies) (Balestrino and Schapira, 2020). Most studies on PD subtypes evaluated biomarkers in the cerebrospinal fluid (CSF) and blood, and the results held promise to define the pathophysiological bases of the different PD subtypes in the future. The CSF levels of α-synuclein, total tau, phosphorylated tau, and beta-amyloid 42 differed in PD patients and healthy controls; however, the attempts to correlate them with clinical symptoms provided inconsistent results. Blood studies showed that higher levels of pro-inflammatory markers were linked with a more severe motor phenotype (IFN-γ, TNF-α, IL-2, and IL-10), faster motor progression (CRP and IL-6), cognitive impairment (IFN-γ, TNF-α, IL-2, and IL-10), and worse sleep quality (CRP). In contrast, higher levels of anti-inflammatory markers (IL-4 and IL-13) correlated with milder motor progression (Williams-Gray et al., 2016; Lawton et al., 2020; Lee et al., 2022). Yet, these studies have potential drawbacks making their application in clinical practice challenging. The concomitant inflammatory process or medications may change the levels of inflammatory markers. Additionally, a routine application of CSF assays to monitor PD patients is challenging (Figura and Friedman, 2020; Mestre et al., 2021; Lee et al., 2022).

In light of this, the genetic markers seem to be the most promising to be used in clinical practice in the future. However, we recognize several issues to be addressed in future research. First, the previous studies used different PD subtyping classifications, which was one of the most significant factors behind the incompatibility of the studies. Therefore, we think there is a strong need to unify the PD subtyping. Of note, the classification should consider the needs of clinicians and should be easy to use in everyday clinical practice. We propose incorporating the subtyping from Konno et al. (2018). Contrary to the two other commonly used classification systems (Zetusky et al., 1985; Jankovic et al., 1990; Schiess et al., 2000; Korchounov et al., 2004; Kang et al., 2005), it is the only one considering AR and GD separately, besides the TD and mixed subtypes.

Secondly, based on our review, previous studies found little overlap between genetic risk factors for PD and genetic modifiers of PD subtypes and progression, and it seems that different genetic loci underlie the susceptibility for PD and its heterogeneity. As lumping them together may impede the research yield, we believe they should be investigated separately. Lastly, the advances in genetic research moved the spotlight from rare and highly penetrant monogenic variants to common variants (polymorphisms) that have low penetrance but a higher impact on the general population. However, despite using more advanced methods and large cohorts, most studies provided results that were not replicated. The failure to implement the findings into clinical practice may be partially caused by a number of studies with often discrepant methods and different results. Therefore, we call for more consistency in the studies by first assessing the known variants’ associations and then investigating the new variants and their links.

As the treatment landscape continues to evolve, precision medicine combining PD subtyping, genetic profile, and biomarkers offers the opportunity to tailor disease treatment to an individual patient. For example, patients with *LRRK2* mutations resulting in a gain of LRRK2 kinase activity are potential candidates for drugs inhibiting its activity or antisense oligonucleotides silencing the altered *LRRK2* gene (Prasuhn and Brüggemann, 2021; Lee et al., 2022). Patients with mitochondrial dysfunction, as with *PRKN* or *PINK1* mutations, are being tested for the potential benefits of coenzyme Q10 or vitamin K2 (Prasuhn and Brüggemann, 2021; Lee et al., 2022). Reduced activity of glucocerebrosidase (GCase) in *GBA* mutation carriers prompted studies aiming at enhancing its activity, its external augmentation, or reduction of its substance (glucosylceramide) (Prasuhn and Brüggemann, 2021; Lee et al., 2022). As LRRK2 activity is elevated in idiopathic PD and GCase can decrease α-synuclein accumulation, these therapies may also be helpful in idiopathic PD patients (Prasuhn and Brüggemann, 2021; Lee et al., 2022).

In summary, the findings from the genetic studies on PD heterogeneity hold promise for utility in clinical practice. However, it is necessary to unify PD subtyping classification and facilitate the link between research and clinical practice. As there is growing evidence that different genetic factors underlie the development of PD and its heterogeneous presentation, we believe the dichotomy is needed to address the unmet needs of research and clinical practice aspects of PD.

## Author contributions

JD took the lead in writing the manuscript. RU and OR revised the manuscript. ZW supervised the preparation of the manuscript and revised it. All authors contributed to the article and approved the submitted version.
